# Cost analysis of antiarrhythmic drug monitoring in patients hospitalized with atrial fibrillation and flutter

**DOI:** 10.1016/j.hroo.2025.03.018

**Published:** 2025-03-24

**Authors:** Abdul Mannan Khan Minhas, Taha Mansoor, Mohamed M. Gad, Mihail G. Chelu, Jalaj Garg, Dmitry Abramov

**Affiliations:** 1 Division of Cardiology, Baylor College of Medicine, Houston, Texas; 2 Section of Cardiovascular Research, Baylor College of Medicine, Houston, Texas; 3 Department of Internal Medicine, Western Michigan University Homer Stryker M.D. School of Medicine, Kalamazoo, Michigan; 4 Division of Cardiovascular Medicine, Loma Linda University Medical Center, Loma Linda, California

**Keywords:** Antiarrhythmic drugs, Atrial fibrillation, Atrial flutter, Cost, Length of stay

**Key Findings**

▪ Hospitalizations for atrial fibrillation/flutter and therapeutic drug monitoring, which may indicate oral antiarrhythmic initiation, are frequent in the United States.
▪ Hospitalizations for atrial fibrillation/flutter and therapeutic drug monitoring cost approximately $1 Billion in the United States from 2016-2019.
▪ Future considerations for faster inpatient protocols or outpatient protocols for oral antiarrhythmic initiation in patients with atrial fibrillation/flutter are needed.

Oral antiarrhythmic drug (AAD) therapy is commonly used for rhythm control in patients with atrial fibrillation or flutter (AF). Among the AADs, sotalol and dofetilide require inpatient admission for drug initiation for QTc monitoring because of their pro-arrhythmic effects. Although guidelines recommend inpatient initiation, recent data have suggested that initiation of AAD outside the hospital is feasible, with a favorable safety profile, particularly in patients with cardiac implantable electronic devices.^1^^,^^2^ Despite differences in clinical practice for AAD initiation, there is an absence of data on the current frequency of hospitalizations with AF and concurrent drug monitoring in the United States. Therefore, we examined the National Inpatient Sample (NIS) to evaluate hospitalizations and costs associated with drug monitoring in the presence of AF between 2016 and 2019.

This was a retrospective analysis of discharge data from the NIS between January 1, 2016 and December 31, 2019. Institutional Review Board approval was not sought because the de-identified database is publicly available via the Healthcare Cost and Utilization Project—NIS. NIS 2016 to 2019 was queried for patient hospitalizations age ≥18 years, using the International Classification of Disease (ICD) 10th procedure code I48.XXX for atrial fibrillation and atrial flutter, and Z51.81 for “Encounter for therapeutic drug level monitoring.” We identified those hospitalizations in which ICD codes for both “Encounter for therapeutic drug level monitoring” and (either) atrial flutter or atrial fibrillation were present in any of the diagnosis fields. All analyses were done using discharge weights provided by the NIS to obtain national estimates. Hospital total charges, inflated to 2021 US dollars, were converted to cost estimates using hospital-specific cost-to-charge ratios provided by the Healthcare Cost and Utilization Project.

Between 2016 and 2019, there were 18,587,224 hospitalizations with AF in the United States, with 59,585 (0.32%) hospitalizations involving a concomitant diagnosis of therapeutic drug monitoring. Data by study year are presented in Table 1. For hospitalizations with both AF and therapeutic drug monitoring coded, the mean length of stay was 5.0 (95% confidence interval [CI], 4.8–5.2) days, the inpatient mortality was 2.6%, and the mean inflation adjusted cost per hospitalization was $17,246 (95% CI, $16,276–$18,215), for a total inflation adjusted cost over the study period of $948,958,249. When therapeutic drug monitoring was evaluated as a primary hospitalization diagnosis with AF in any of the other fields, only 1980 cases over the 4-year study period were noted, with mean length of stay of 3.2 (95% CI, 3.0–3.4) days and the mean inflation adjusted cost per hospitalization of $5612 (95% CI, $5,028.9–$6,194.3).

**Table 1:** Encounter for therapeutic drug level monitoring for atrial flutter or atrial fibrillation

	2016	2017	2018	2019
Total hospitalizations with atrial fibrillation	4,354,937	4,591,413	4,750,794	4,890,080
Hospitalizations with atrial fibrillation and therapeutic drug level monitoring	12,475	15,060	16,425	15,625
Percentage of hospitalizations with therapeutic drug monitoring	0.29%	0.33%	0.35%	0.32%
Length of stay for drug monitoring hospitalization	5.2	5.1	4.9	4.9
Mean costs of drug monitoring hospitalizations	$13,943	$18,129	$17,599	$17,763

Our findings suggest that hospitalizations with AF and therapeutic drug monitoring remain frequent in the current era, with approximately sixty thousand hospitalizations costing almost 1 billion dollars in the United States between 2016 and 2019. Although guidelines mandate inpatient monitoring for sotalol and dofetilide initiation because of high risk of life-threatening ventricular arrhythmias until steady state is achieved, recent studies have suggested that outpatient initiation of sotalol using lower starting doses and protocolized outpatient monitoring is associated with a favorable safety profile.^1^ Other prior studies on outpatient initiation of AAD therapy in patients with preexisting cardiac implantable electronic devices or with noninvasive rhythm monitoring devices may allow further clinician comfort in the outpatient setting. Alternatively, studies also have demonstrated that sotalol can be rapidly loaded via an intravenous formulation to achieve steady state—thereby overcoming a 3-day or greater hospitalization and resulting in significant cost savings.^3^^,^^4^

Our findings have important limitations. The data are based on ICD codes, and likely many of the hospitalizations with AF and therapeutic drug monitoring did not primarily occur for therapeutic drug monitoring. Although the number of patients hospitalized with AF in which therapeutic drug monitoring was the primary diagnosis is presented, this likely significantly underestimates the true prevalence of antiarrhythmic therapy initiation, given the high number of patients in the United States currently receiving antiarrhythmic therapy.^5^ The specific medication that required therapeutic drug monitoring is not available within the dataset. However, we show that the therapeutic drug monitoring code is used in a small percentage of patients with hospitalization with AF, which suggests that therapeutic drug monitoring less likely refers to anticoagulation monitoring in this setting.

In conclusion, we demonstrate that admissions with AF and therapeutic drug monitoring are common in the United States and are associated with significant system costs. These data may provide an opportunity to revisit current guidelines and standard of care for initiation of certain classes of AAD therapies in the setting of AF.
